# Resting State Functional Connectivity Among Cortical and Subcortical Grey Matter Structures Can Distinguish Fallers in Parkinson's Disease

**DOI:** 10.1111/ejn.70666

**Published:** 2026-08-27

**Authors:** Rodrigo Vitorio, Korey P. Wylie, Justin W. Andrushko

**Affiliations:** ^1^ School of Sport, Exercise and Rehabilitation, Faculty of Health and Wellbeing Northumbria University Newcastle upon Tyne UK; ^2^ Department of Psychiatry University of Colorado Anschutz Medical Campus Aurora Colorado USA

**Keywords:** balance, falls, gait, magnetic resonance imaging

## Abstract

The underlying neural mechanisms associated with falls in Parkinson's disease (PD) are not fully understood. We aimed to identify measures of resting‐state functional connectivity (rs‐FC) among cortical and subcortical regions that best discriminate fallers from nonfallers in people with PD. People with mild‐to‐moderate PD were classified as fallers (*n* = 23) or nonfallers (*n* = 35) based on falls in the previous 12 months. Following resting‐state functional magnetic resonance imaging, measures of rs‐FC were obtained for multiple subcortical and cortical regions of interest (ROIs) associated with the control of gait and balance. To investigate the combinations of (up to three) measures that best discriminate fallers from nonfallers, we applied logistic regression employing a ‘best subsets selection strategy’ with a five‐fold cross validation and calculated the area under the curve (AUC). The most consistently selected measures in the top 10 ranked models were the rs‐FC between the following ROIs: left dorsolateral prefrontal cortex (DLPFC)–hypothalamus, and somatosensory cortex–globus pallidus internus. Relative to nonfallers, fallers had reduced rs‐FC between these two ROIs pairs (Cohen's *d* = 0.918 and 0.765, respectively). The model combining these pairs with the rs‐FC between the right DLPFC and the thalamus achieved an AUC of 0.87 (95% CI: 0.77–0.97). The rs‐FC between the left DLPFC and the hypothalamus, and between the somatosensory cortex and the globus pallidus internus, discriminate fallers from nonfallers in PD, representing potential targets for interventions. Connectivity deficits may limit the ability of fallers in processing information in a timely and accurate manner.

## Graphical abstract

In 58 people with Parkinson's disease, fallers showed weaker resting‐state connectivity between the left dorsolateral prefrontal cortex and hypothalamus, and between somatosensory cortex and globus pallidus internus. Combining these measures discriminated fallers from nonfallers, identifying potential targets for fall prevention. The figure was created with Biorender.com.

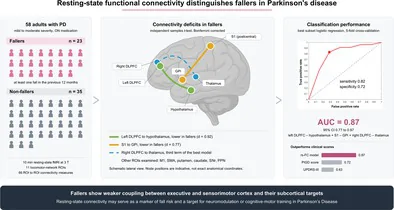

**Abbreviations**
ASSET: array spatial sensitivity encoding technique
AUC: area under the curve
BET: brain extraction tool
BGHAT: Basal Ganglia Human Area Template
BIC: Bayesian Information Criteria
CI: confidence interval
DLPFC: dorsolateral prefrontal cortex
FAST: FMRIB's Automated Segmentation Tool
FC: functional connectivity
FIRST: FMRIB's Integrated Registration and Segmentation Tool
FIX: FMRIB's ICA‐based Xnoiseifier
FLIRT: FMRIB's Linear Image Registration Tool
fMRI: functional magnetic resonance imaging
FNIRT: FMRIB's Nonlinear Image Registration Tool
FOV: field of view
FSL: FMRIB Software Library
GPi: globus pallidus internus
ICA: independent component analysis
LEDD: levodopa equivalent daily dose
M1: primary motor cortex
MCFLIRT: Motion Correction FMRIB's Linear Image Registration Tool
MNI: Montreal Neurological Institute
MoCA: Montreal Cognitive Assessment
MRI: magnetic resonance imaging
NPV: negative predictive value
PD: Parkinson's disease
PFC: prefrontal cortex
PIGD: postural instability and gait difficulty
PPN: pedunculopontine nucleus
PPV: positive predictive value
ROI: region of interest
rs‐FC: resting‐state functional connectivity
S1: somatosensory cortex
SD: standard deviation
SMA: supplementary motor area
SNr: substantia nigra pars reticulata
SPGR: spoiled gradient recalled echo
T1w: T1‐weighted
TE: echo time
TR: repetition time
UPDRS‐III: Unified Parkinson's Disease Rating Scale, Part III

## 1 Introduction

Falls are a major concern among people with Parkinson's disease (PD), affecting approximately 60% of individuals annually (Allen et al. 2013). Falls lead to serious physical, psychological and economic consequences, including fractures, hospitalization, loss of independence and increased healthcare costs (Paul et al. 2017; Florence et al. 2018; Rudzińska et al. 2013). Identifying the neural mechanisms underlying falls is critical for developing targeted interventions to reduce fall risk and improve quality of life in people with PD. However, despite the well‐documented multifactorial nature of falls in PD (Fasano et al. 2017; Vitorio, Mancini, et al. 2023; Araújo et al. 2023), including gait and balance impairments and other aspects such as impaired cognition, the specific neural pathways associated with falls are still not fully understood.

Neuroimaging research has identified the involvement of several cortical and subcortical regions in gait and balance control, including two distinct corticospinal networks (Hamacher et al. 2015; la Fougere et al. 2010; Herold et al. 2017). The automatic locomotor network, primarily engaged during steady‐state walking, involves direct connections from the primary motor cortex (M1) to central pattern generator circuits in the spinal cord (Hamacher et al. 2015; la Fougere et al. 2010). In contrast, the executive locomotor network becomes active during complex walking tasks that require planning and modulation of locomotion (Fasano et al. 2017; la Fougere et al. 2010). In clinical cohorts with impaired gait automaticity (including PD), the executive locomotor network is activated even during steady‐state walking as a compensatory mechanism. Within the executive locomotor network, signals originate from the supplementary motor area (SMA) and prefrontal cortex (PFC), travelling through the basal ganglia (including the striatum, pallidum and subthalamic nucleus) and the mesencephalic locomotor region before reaching the medullary and pontine reticular formations in the brainstem, which send projections down the spinal cord (Florence et al. 2018; la Fougere et al. 2010). Additionally, the somatosensory cortex (S1) plays a critical role in gait and balance control by integrating afferent sensory inputs from the periphery to provide feedback on body position, movement and contact with the ground. Consequently, dysfunction at the above‐mentioned cortical and/or subcortical brain regions could impair gait and balance control, thereby leading to a greater risk of falls.

In PD, both pathophysiological processes and compensatory mechanisms for the control of gait and balance have been reported. For example, we recently showed that, compared to healthy controls, PD leads to a greater proportion of low‐frequency neuronal firing in the sensorimotor cortex while walking, representing a potential marker of impaired automaticity (Vitório, Lirani‐Silva, et al. 2023). Consequently, the executive locomotor network is activated as a compensatory mechanism, even during simple walking tasks (Orcioli‐Silva et al. 2021; Orcioli‐Silva et al. 2020; Maidan et al. 2015). Executive‐attentional resources are thought to be needed to compensate for deficits in movement automaticity in people with PD (Orcioli‐Silva et al. 2021; Orcioli‐Silva et al. 2020; Maidan et al. 2015). These pathophysiological processes and compensatory mechanisms lead to changes in how cortical and subcortical brain regions interact with each other during gait and balance tasks in people with PD. Therefore, investigating measures of functional connectivity (FC) between key cortical and subcortical brain regions involved in gait and balance control may enhance our understanding of the neural mechanisms underlying falls in PD.

Resting‐state functional connectivity (rs‐FC), measured with functional magnetic resonance imaging (fMRI), offers a powerful approach for studying neural network interactions and their association with movement, balance and even falls with excellent spatial resolution and the ability to measure deep brain structures (Ragothaman et al. 2022). To date, only a few studies have explored the link between rs‐FC and falls in PD. Rosenberg‐Katz et al. (2015) reported increased rs‐FC between parietal nodes of the central executive network in PD fallers compared to nonfallers and healthy controls, with no changes in rs‐FC within the sensorimotor network. The observed decrease in rs‐FC within the central executive network supports the interpretation that attention and executive function contributes to falls in people with PD (Rosenberg‐Katz et al. 2015). Additionally, Otomune et al. found reduced rs‐FC between the left pallidum and the right perisylvian region and postcentral gyrus in people with PD who were frequent fallers (Otomune et al. 2019). However, to date no research has focused on rs‐FC analyses in regions within the automatic and executive locomotor networks for association with falls in PD. Therefore, we explored measures of rs‐FC within the automatic and executive locomotor networks to discriminate fallers from nonfallers among people with mild‐to‐moderate PD.

This study aimed to identify measures of rs‐FC between key cortical and subcortical regions, involved in gait and balance control, that best discriminate fallers from nonfallers in people with mild‐to‐moderate PD. Based on prior evidence of executive network dysfunction in PD fallers (Rosenberg‐Katz et al. 2015) and the role of sensorimotor integration in balance control (Gorst et al. 2019), we hypothesised that: (1) fallers would show reduced rs‐FC between dorsolateral prefrontal cortex (DLPFC) and subcortical motor regions involved in locomotor control, reflecting impaired executive modulation of movement; and (2) fallers would show reduced rs‐FC between sensory (S1) and motor regions (basal ganglia), reflecting compromised sensorimotor integration. Given the multifactorial nature of falls in PD, we further hypothesised that a combination of rs‐FC measures would best discriminate fallers from nonfallers. We employed a data‐driven best subset selection approach with logistic regression and cross‐validation to identify which specific connectivity patterns within these networks best discriminate fallers from nonfallers. Findings may provide critical insight into the neural mechanisms underlying falls in PD and highlight potential targets for therapeutic interventions.

## 2 Methods

### 2.1 Design and Participants

This is a cross‐sectional study exploring a dataset made publicly available at https://openneuro.org/datasets/ds004392/versions/1.0.0 (Wylie et al. 2026, 2023). The dataset contains structural and resting‐state functional MRI scans from 58 patients with PD who were recruited from the University of Colorado Hospital's Movement Disorder, Memory Disorder and Neuropsychology Clinics (Wylie et al. 2023). All participants had a confirmed diagnosis of PD based on the UK Brain Bank Criteria (Hughes et al. 1992) and were on stable medications for a minimum of 30 days. Exclusion criteria were the following: indicators suggestive of other causes of parkinsonism or Parkinson‐plus syndromes; indicators suggestive of other causes of dementia, including moderate to severe cerebrovascular disease (history or imaging); history of deep brain stimulation, ablation surgery or other brain surgery; history of major head trauma; evidence for moderate depression based on the Hospital Anxiety Depression Scale (score > 11; Zigmond and Snaith 1983),; general MRI exclusion factors (e.g., claustrophobia, body weight > 136 kg, metal in the body). All participants had less than 2 mm or 2° movement in any direction (Wylie et al. 2023). The study was approved by the Colorado Multiple Institution Review Board and adheres to the ethical standards of the Declaration of Helsinki. Participants gave informed consent to participate.

### 2.2 Clinical Assessment

Participants underwent a clinical assessment to obtain the following information: sex, age, years of education, disease/symptoms severity [Unified Parkinson's Disease Rating Scale (UPDRS) part III], disease stage [Hoehn & Yahr Rating Scale (H&Y)], global cognition score [Montreal Cognitive Assessment (MoCA)], levodopa equivalent daily dose (LEDD) and history of falls (self‐report). Participants were classified as fallers (i.e., one or more falls) or nonfallers (i.e., no falls) based on falls sustained in the previous 12 months. Fall was defined as an unintentionally coming to the ground or some lower level not as a result of a major intrinsic event (e.g., stroke) or overwhelming hazard (Vitorio, Mancini, et al. 2023; Araújo et al. 2023).

### 2.3 MRI Acquisition

Participants were tested in their usual ON medication state. Images were acquired on a 3 T Signa scanner (General Electric, Milwaukee), using an 8‐channel head coil and a 3‐D, extended dynamic range, inversion recovery SPGR ASSET parallel imaging sequence. T1‐weighted (T1w) structural scans were acquired with the following parameters: repetition time (TR) = 2200 ms, echo time (TE) = 2 ms, matrix = 256 × 256, field of view (FOV) = 256 × 256 mm^2^, voxel size = 1 × 1 mm^2^, slice thickness = 1 mm, flip angle = 8°. Functional MRI scans were acquired with the following parameters: TR = 2000 ms, TE = 26 ms, matrix = 64 × 64, FOV = 218 × 218 mm^2^; voxel size = 3.4 × 3.4 mm^2^, slice thickness = 3.5 mm, gap = 1.5 mm, flip angle = 70°, with an interleaved acquisition. A total of 10 min of resting state scanning was recorded. MRI images were acquired within 1 day of the clinical testing.

### 2.4 MRI Data Processing—Structural

The T1w structural MRI scans were processed using FMRIB Software Library (FSL) tools (Jenkinson et al. 2012; Smith et al. 2004; Woolrich et al. 2009). First, *fslreorient2std* was used to reorient the images to match standard‐space images, then *robustfov* was used to crop the image in the Z‐direction to remove the neck from the images. Brain extraction was carried out using the brain extraction tool (BET) (Smith 2002). Tissue type segmentation was completed using FAST (Zhang et al. 2001), and FIRST (Patenaude et al. 2011) was used to perform subcortical segmentation. At each step in the processing pipeline data were visually inspected to ensure the brains were extracted and segmented correctly.

### 2.5 MRI Data Processing—Functional

The functional scans were pre‐processed using melodic with the following settings: motion correction was applied using MCFLIRT, 100‐s high pass filter, *BET* for functional images and finally no spatial smoothing was applied to preserve the spatial features of subcortical brain regions we aimed to measure. The registration matrix and warps were calculated using a linear registration of the functional data to their respective T1w native‐space structural scan using *FLIRT* (Jenkinson et al. 2002; Jenkinson and Smith 2001) and nonlinearly registered to MNI‐152 standard‐space with FNIRT (Smith et al. 2004). After these minimal pre‐processing steps were completed, an unconstrained independent component analysis (ICA) data decomposition was performed on the subject‐level data. Ten scans were used to train ICA‐based Xnoiseifier (FIX) (Griffanti et al. 2014; Salimi‐Khorshidi et al. 2014) to create a custom component classifier, and *ICA‐FIX* was then carried out to perform automatic component classifications, labelling components into signal or noise. Noise components were removed using a FIX classification threshold of 30. In the same cleanup step, the ‘‐m’ option was applied to regress out motion confounds (i.e., the 24 standard motion parameters [the six rigid‐body parameters, their temporal derivatives and the squares of both]), which were highpass filtered at 100 s to match the temporal filtering applied to the functional data. Manual inspection of the *ICA‐FIX* results was carried out for all scans to ensure classification accuracy.

After ICA data cleaning, the functional scans were registered to standard‐space using the *applywarp* command, using the previously defined linear and nonlinear registration matrix and warp files.

Regions of interest (ROIs) included the following brain regions involved in the executive and/or automatic locomotor network (Hamacher et al. 2015; la Fougere et al. 2010) as well as the output nuclei of the basal ganglia (Lanciego et al. 2012): M1, S1, DLPFC (treated as left and right DLPFC given the functional lateralization of the DLPFC (Kaller et al. 2011)), SMA, thalamus, hypothalamus, putamen, caudate, globus pallidus internus (GPi), substantia nigra pars reticulata (SNr) and pedunculopontine nucleus (PPN). The following atlas was used: Harvard‐Oxford Cortical (Desikan et al. 2006; Frazier et al. 2005; Goldstein et al. 2007; Makris et al. 2006), Brainnetome (Fan et al. 2016), BGHAT (Prodoehl et al. 2008) and Brainstem Navigator Toolkit (Bianciardi et al. 2018; Bianciardi et al. 2015; García‐Gomar et al. 2019; García‐Gomar et al. 2022; Singh et al. 2021; Singh et al. 2019). The hypothalamus ROI was drawn manually, as no suitable atlas mask was available. Only the DLPFC was retained as separate left and right ROIs. For M1, S1, thalamus, hypothalamus, putamen, caudate, GPi, SNr and PPN, the atlas provided a single mask spanning both hemispheres, whereas for the SMA separate left and right masks were merged into a single bilateral mask. Each ROI (Table 1) was registered to the 2 mm standard‐space template using *flirt*, thresholded and binarized using *fslmaths*. Then, the ROIs were concatenated into a four‐dimensional file using *fslmerge* followed by *slice_summary* to create thumbnail images for each ROI.

**TABLE 1:** Regions of interest.

Region of interest	Acronym	Hemisphere		Atlas
		Left	Right	
Primary motor cortex	M1	Bilateral		Harvard‐Oxford Cortical (Desikan et al. 2006; Frazier et al. 2005; Goldstein et al. 2007; Makris et al. 2006)
Primary somatosensory cortex	S1	Bilateral		Harvard‐Oxford Cortical
Dorsolateral prefrontal cortex	DLPFC	✔	✔	Brainnetome (Fan et al. 2016)
Supplementary motor area	SMA	Bilateral (merged)		Brainnetome
Thalamus		Bilateral		Brainnetome
Hypothalamus		Bilateral		Custom drawn
Putamen		Bilateral		Harvard‐Oxford Subcortical
Caudate		Bilateral		BGHAT (Prodoehl et al. 2008)
Globus pallidus internus	gpi	Bilateral		BGHAT
Substantia nigra pars reticulata	SNr	Bilateral		Brainstem Navigator Toolkit (Bianciardi et al. 2018; Bianciardi et al. 2015; García‐Gomar et al. 2019; García‐Gomar et al. 2022; Singh et al. 2021; Singh et al. 2019)
Pedunculopontine nucleus	PPN	Bilateral		Brainstem Navigator TOOLKIT

*Note:* Check marks indicate ROIs for which separate left and right masks were retained and analysed as distinct ROIs. ‘Bilateral’ indicates a single atlas mask spanning both hemispheres. ‘Bilateral (merged)’ indicates separate left and right masks that were combined into a single bilateral mask.

The mean timeseries of each ROI in standard‐space was extracted using *fslmeants*, creating a subject and ROI specific text file. Next, an ROI matrix was created for each subject by combining the individual ROI timeseries into a single file, with each column representing a different ROI's timeseries. These timeseries matrices were then used to calculate ROI‐ROI functional connectivity using FSLNets.

### 2.6 Functional Connectivity Analysis

To analyse the rs‐fMRI data, FSLNets was used to carry out network modelling. The subject‐level timeseries matrices were fed into FSLNets to calculate a network matrix for each subject. FSLNets calculates full and partial correlations for each edge of the network matrix (i.e., connectivity between each ROI pairing). The partial correlation matrix was extracted and carried forward for subsequent group level analyses in JASP (Love et al. 2019).

### 2.7 Statistical Analysis

To investigate which ROI‐ROI measures of rs‐FC or combinations of them that best discriminate fallers from nonfallers, we performed logistic regression employing a best subset selection strategy (as feature selection method) and five‐fold cross validation. This approach was previously employed by Vitorio and colleagues (Vitorio, Mancini, et al. 2023), using clinical and mobility measures to classify fallers and nonfallers. Our dataset consisted of 66 connectivity measures derived from fMRI, and we applied a systematic search strategy testing combinations of one, two and three measures (due to our sample size). For each combination, we trained logistic regression models using five‐fold cross‐validation to assess model performance. The data was split in five parts such that 80% randomly selected individuals were used for feature selection and training, whereas the remaining 20% were used for validation. The best subset selection strategy is recommended since various combinations of measures are possible with similar classification results. It selects the best model from all possible subsets according to model complexity and goodness‐of‐fit criteria, which was assessed using the Bayesian Information Criteria (BIC) (Hosmer et al. 2013). We selected the top 10 models based on BIC (i.e., lowest BIC values) and computed five‐fold cross‐validation area under the receiver operating characteristic curve (AUC). An AUC ≥ 0.9 is considered outstanding, between 0.8 and 0.9 is considered excellent, and between 0.7 and 0.8 is considered acceptable (Hosmer et al. 2013). The analysis was performed using MATLAB version R2021a (Mathworks, Natick, MA, USA). Given our sample size (23 fallers, 35 nonfallers), we limited combinations to a maximum of three measures and relied on BIC, which penalizes model complexity, together with cross‐validated (rather than in‐sample) performance evaluation, to guard against overfitting. Considering the top 10 best‐fitting models, rather than a single selected model, further reduced the risk of overfitting to idiosyncrasies of any one model.

Additionally, we looked at the importance of a particular measure of rs‐FC from top 10 ranked models incorporating measures selected by best subset selection. Most frequently selected measures were then compared between fallers and nonfallers using the appropriate test (independent sample *t*‐test or Mann–Whitney test) according to the data distribution (Shapiro–Wilk test). The alpha level was set to 0.05 for statistical testing, with Bonferroni correction for multiple comparisons. For comparison with the rs‐FC models, receiver operating characteristic analyses were also performed for the UPDRS‐III total score and for the postural instability and gait difficulty (PIGD) score derived from the UPDRS. The independent sample *t*‐test and the Mann–Whitney test were implemented in SPSS version 26 (The International Business Machines Corporation, USA).

## 3 Results

### 3.1 Participants

Twenty‐three patients were classified as fallers (39.7%; including 10 recurrent fallers) and 35 as nonfallers (60.3%). Demographic characteristics of fallers and nonfallers are shown in Table 2. Overall, participants had mild to moderate disease severity. Fallers and nonfallers were not statistically different for age, sex, years of education or global cognition (MoCA); fallers tended to have worse motor symptoms (UPDRS‐III, Table 2).

**TABLE 2:** Participants characteristics.

Variables	Combined mean (SD)	Fallers mean (SD)	Nonfallers mean (SD)	*p*
Sex (male/female)	M = 39, F = 19	M = 16, F = 7	M = 23, F = 12	0.760
Age (years)	70.4 (7.9)	71.1 (8.4)	69.8 (7.4)	0.541
Education (years)	16.2 (2.7)	16.9 (1.8)	15.8 (3.2)	0.138
UPDRS III (0–108 score)	25.2 (9.2)	27.9 (9.0)	23.4 (9.0)	0.068
PIGD (score)	2.7 (2.5)	4.0 (2.9)	1.9 (1.7)	0.005
LEDD (mg/day)	530.3 (431.8)	597.9 (429.2)	485.8 (434.0)	0.258
MoCA (0–30 score)	24.5 (4.3)	23.4 (4.8)	25.2 (3.8)	0.118

### 3.2 Functional Connectivity in Discriminating Fallers and Nonfallers

**FIGURE 1:**
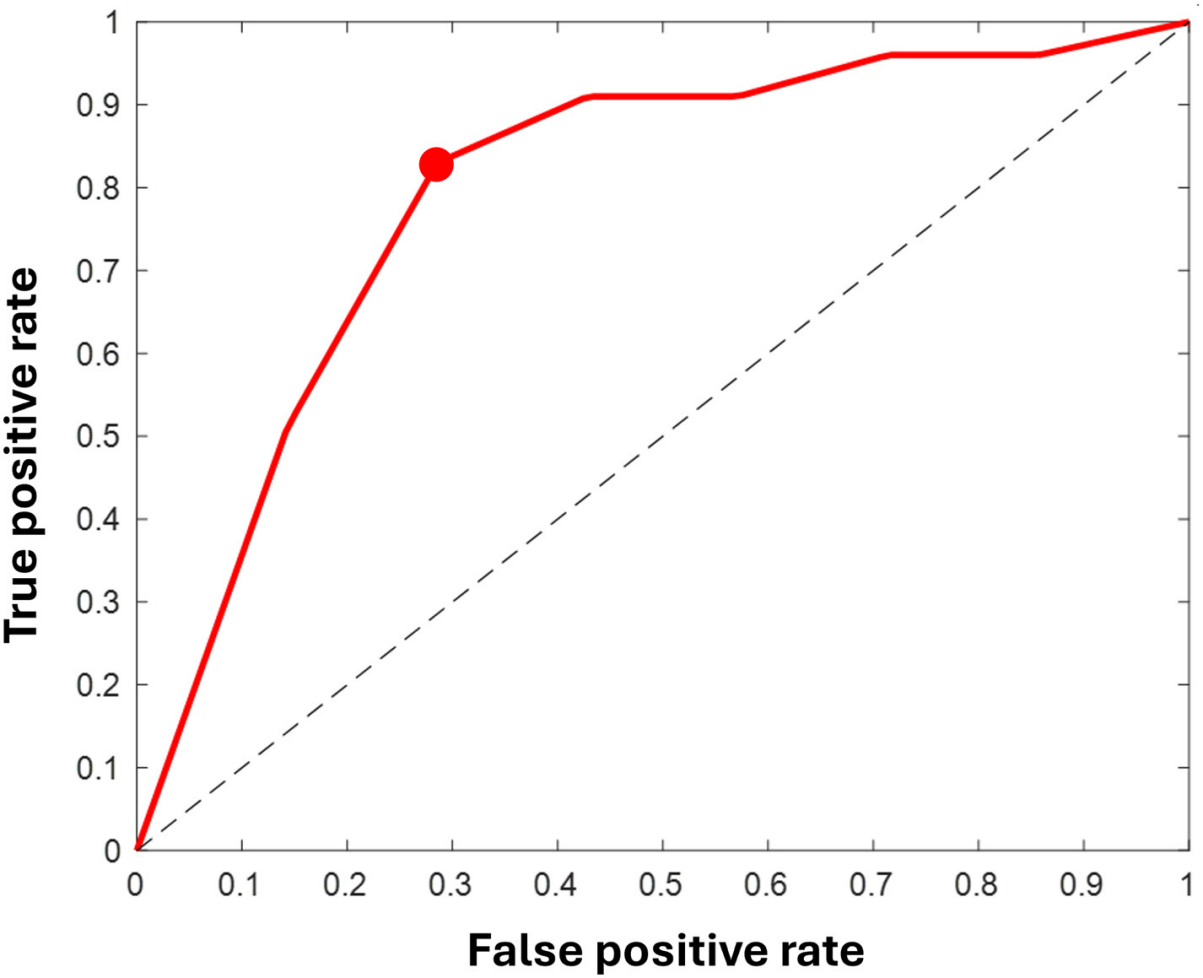
Receiver operating characteristic curve for the model with the highest AUC (i.e., left DLPFC–hypothalamus, S1–GPi, and right DLPFC–thalamus) in discriminating fallers from nonfallers in PD. The point closest to the (0,1) corner had a sensitivity of 0.824 and a specificity of 0.717.

The top 10 ranked models in discriminating fallers and nonfallers in PD are shown in Table 3. Nine models had AUCs corresponding to excellent discrimination. The model with the highest AUC (0.87; 95% CI: 0.77–0.97) included measures of rs‐FC between the following ROIs: left DLPFC–hypothalamus, S1–GPi and right DLPFC–thalamus (Table 3). Such model had a sensitivity of 0.824, a specificity of 0.717, a positive predictive value (PPV) of 0.66, and a negative predictive value (NPV) of 0.86 in discriminating fallers and nonfallers (Figure 1). For comparison, UPDRS‐III (AUC = 0.629, *p* = 0.100) and the postural instability and gait difficulty (PIGD) score (AUC = 0.718, *p* = 0.005) performed worse than the rs‐FC models in discriminating fallers and nonfallers.

**TABLE 3:** Top 10 ranked models (based on BIC) in discriminating fallers and nonfallers in PD. ROI pairs that repeat across the models are coloured to facilitate their identification.

Measures of functional connectivity						BIC	AUC	95% CI	PPV	NPV
1st		2nd		3rd						
	S1‐GPi		Left DLPFC‐Hypothalamus		Right DLPFC‐Thalamus	56.02	0.87	0.77–0.97	0.66	0.86
	S1‐GPi		Left DLPFC‐Hypothalamus		Hypothalamus‐SNr	54.26	0.86	0.75–0.97	—	—
	S1‐Caudate		S1‐GPi		Left DLPFC‐Hypothalamus	55.04	0.86	0.75–0.97	—	—
	S1‐GPi		Left DLPFC‐Hypothalamus			55.94	0.85	0.74–0.96	—	—
	S1‐GPi		SMA‐Caudate		Left DLPFC‐Hypothalamus	57.30	0.84	0.73–0.95	—	—
	M1‐Caudate		S1‐GPi		Left DLPFC‐Hypothalamus	57.10	0.83	0.72–0.94	—	—
	Left DLPFC‐Hypothalamus		Left DLPFC‐Putamen		Hypothalamus‐SNr	57.18	0.83	0.72–0.94	—	—
	S1‐GPi		Left DLPFC‐Hypothalamus		Putamen‐SNr	57.35	0.83	0.72–0.94	—	—
	S1‐PPN		Left DLPFC‐Hypothalamus		Hypothalamus‐SNr	56.30	0.82	0.70–0.94	—	—
	S1‐GPi		Left DLPFC‐Hypothalamus		Left DLPFC‐Putamen	57.13	0.79	0.66–0.92	—	—

Abbreviations: DLPFC: dorsolateral prefrontal cortex; GPi: globus pallidus internus; M1: primary motor cortex; S1: primary somatosensory cortex; SMA: supplementary motor area; SNr: substantia nigra pars reticulata.

**FIGURE 2:**
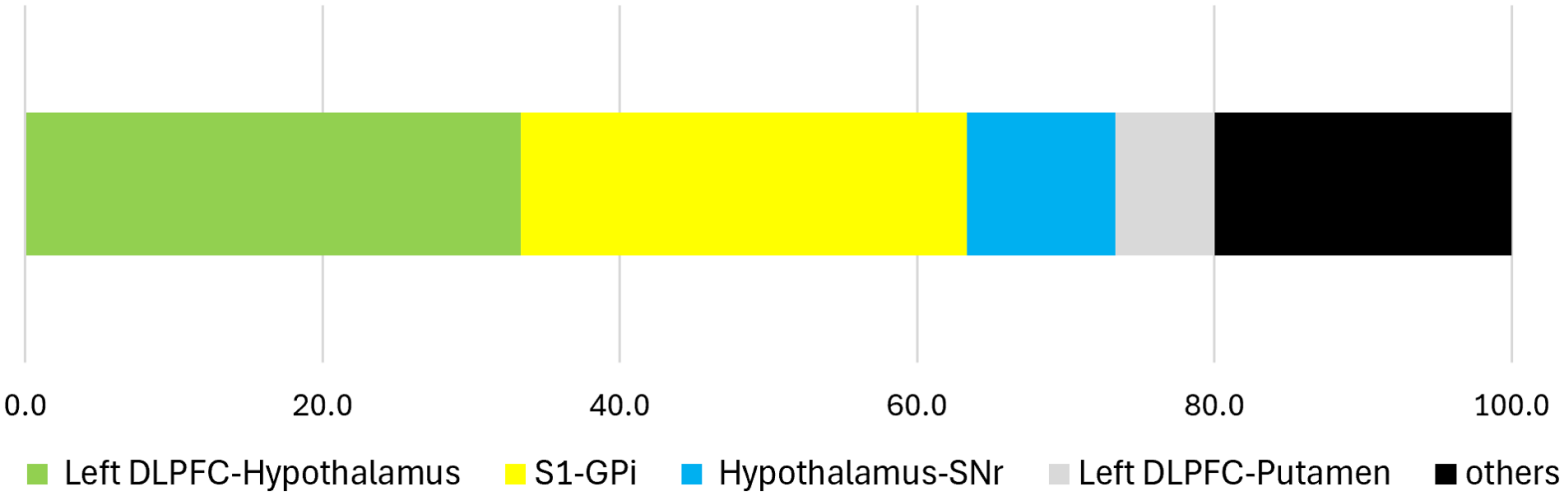
Feature relative importance (most consistently selected measures of functional connectivity) among the top 10 ranked models in discriminating fallers from nonfallers.

The most consistently selected measures in the top 10 ranked models (Figure 2) were the rs‐FC between the following ROIs: left DLPFC–hypothalamus (10×), S1–GPi (9×), hypothalamus–SNr (3×) and left DLPFC–putamen (2×).

**FIGURE 3:**
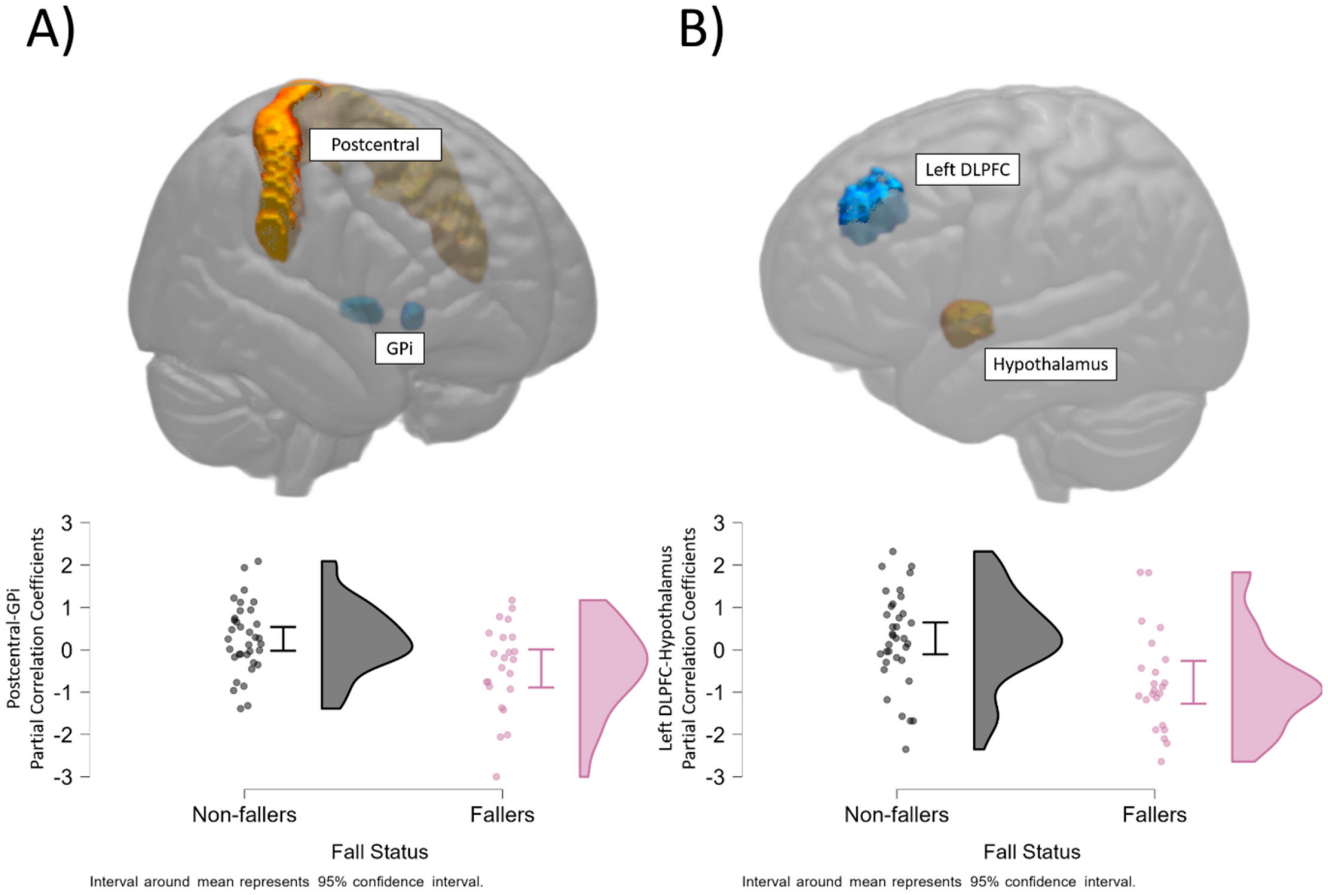
Significant differences between fallers and nonfallers (independent samples *t*‐test, Bonferroni‐corrected *p* < 0.05). (A) Fallers had reduced functional connectivity between the primary somatosensory cortex (S1; labelled ‘Postcentral’ in the figure) and the globus pallidus internus (GPi) and (B) between the left dorsolateral prefrontal cortex (DLPFC) and the hypothalamus.

Relative to nonfallers, fallers had reduced functional connectivity between the following ROI pairs (Figure 3): Left DLPFC–hypothalamus (*t* = 3.420, *p* = 0.001, *p*_Bonferroni_ = 0.004, Cohen's *d* = 0.918 [95% Confidence Interval: 0.362–1.467]) and S1–GPi (*t* = 2.851, *p* = 0.006, *p*_Bonferroni_ = 0.024, Cohen's *d* = 0.765 [95% Confidence Interval: 0.217–1.307]). Fallers and nonfallers had similar functional connectivity between the following ROI pairs: Hypothalamus–SNr (U = 298, *p* = 0.097, *p*_Bonferroni_ = 0.388) and left DLPFC–putamen (*t* = 2.016, *p* = 0.049, *p*_Bonferroni_ = 0.196).

## 4 Discussion

In this study, we built models combining measures of rs‐FC within the locomotor networks to discriminate fallers from nonfallers among people with mild‐to‐moderate PD. As hypothesised, the top performing models included a combination of rs‐FC measures involving regions implicated in the processing of sensorimotor and cognitive information. Fallers had reduced rs‐FC between the S1 and GPi (Figure 3A) and between the left DLPFC and hypothalamus (Figure 3B). These connectivity deficits may reflect impaired integration of executive and sensorimotor signals required for maintaining gait and balance, ultimately contributing to an increased risk of falls.

The left DLPFC is a critical hub within the executive locomotor network, supporting higher‐order functions such as executive function, attention and motor modulation. Connectivity deficits with the hypothalamus, a region involved in autonomic and motivational aspects of locomotion (Takakusaki 2017), may be essential for seamless coordination between cognitive and motor systems during walking. Reduced connectivity between the DLPFC and the hypothalamus could indicate a diminished ability to integrate executive‐attentional resources with motor control, potentially impairing adaptive responses to environmental challenges and increasing fall risk. Similarly, the reduced connectivity between S1 and GPi may compromise the integration of sensory feedback with motor commands (Wang et al. 2024; Permezel et al. 2023), hindering the ability to adjust gait and balance in response to perturbations. This aligns with prior evidence that sensory processing deficits are associated with balance impairments and falls in PD (Gorst et al. 2019).

The model combining these two connectivity measures with the connectivity between the right DLPFC and the thalamus achieved excellent discriminatory performance (AUC = 0.87), underscoring the multifactorial nature of falls in PD. The thalamus serves as a relay for sensory and motor information (Sherman and Guillery 2002; Cabrera‐Álvarez et al. 2023), and its connectivity with the DLPFC likely plays a role in integrating sensory input with executive control for movement planning (Worden et al. 2021). Together, these findings reinforce the notion that disruptions in both the automatic and executive locomotor networks are implicated in PD‐related falls (Vitório, Lirani‐Silva, et al. 2023; Orcioli‐Silva et al. 2021; Orcioli‐Silva et al. 2020; Maidan et al. 2015). Specifically, deficits in connectivity between regions mediating sensory‐motor integration and executive modulation may hinder the ability to process information in a timely and accurate manner, leading to compromised gait and balance control.

Our results add to the growing body of evidence that rs‐FC analyses can provide valuable insights into the neural mechanisms underlying gait and balance dysfunction in PD. Previous studies have reported alterations in rs‐FC within the sensorimotor network and central executive network in PD fallers, but no prior work has systematically investigated connectivity within locomotor networks.

### 4.1 Clinical Implications

The identification of specific FC deficits associated with falls offers potential targets for therapeutic interventions, including neuromodulation techniques (e.g., transcranial magnetic/electrical stimulation and deep brain stimulation) or cognitive‐motor training aimed at enhancing connectivity between ROI pairs with reduced connectivity. Another clinical implication of current findings refers to the use of rs‐FC measures as potential markers (still requiring additional validation in larger samples and prospective studies) of fall risk in people with PD.

The best‐performing model showed strong sensitivity (0.824) and NPV (0.86), but more moderate specificity (0.717) and PPV (0.66), meaning that approximately 28% of nonfallers in our sample would have been misclassified as fallers. These parameters suggest that rs‐FC measures may be particularly useful as a rule‐out tool (NPV higher than PPV), helping to identify individuals unlikely to be at risk of falls, while a positive classification alone would warrant further clinical assessment rather than being treated as confirmatory. It is worth noting that the proportion of fallers in our sample (39.7%) was somewhat lower than the ~60% fall prevalence often reported in PD (Allen et al. 2013). Because PPV increases and NPV decreases with rising outcome prevalence, our reported PPV may modestly underestimate, and our NPV may modestly overestimate, performance in populations with a higher rate of fallers. Rather than functioning as a standalone diagnostic tool, FC measures may be most clinically useful when combined with existing clinical and mobility‐based (gait and balance) assessments. Future work should evaluate whether incorporating rs‐FC alongside established clinical and mobility measures improves specificity and PPV beyond what any single domain achieves alone, and should validate these combined models in larger, prospectively followed cohorts. Notably, measures of rs‐FC performed better than the UPDRS‐III in classifying fallers and nonfallers.

### 4.2 Limitations

Nevertheless, this study has limitations that are important to consider. First, the cross‐sectional design precludes causal inferences about the relationship between rs‐FC deficits and falls. Longitudinal studies are needed to determine whether these connectivity patterns can predict future falls. Second, the classification of fallers and nonfallers was based on self‐reported history of falls, which may be influenced by recall bias. Third, rs‐FC measures may not fully capture the neural dynamics of falls. Fourth, given that all scans were acquired only in the ON‐medication state, our findings cannot be generalized to the OFF state; to date, dopaminergic therapy is known to alter rs‐FC, particularly in prefrontal–basal ganglia networks (Gilat et al. 2017). Consequently, the present findings may reflect a combination of intrinsic disease‐related alterations and medication‐induced changes in network dynamics. Future studies should consider evaluating rs‐FC in both ON and OFF medication conditions to better isolate connectivity features intrinsically linked to fall susceptibility. Fifth, the absence of gait and balance measures limits the clinical characterization of our sample. Sixth, the inclusion of only mild‐to‐moderate PD patients limits the generalisability of our findings to those with more advanced disease. Seventh, ROI selection was constrained a priori to nodes of the executive and automatic locomotor networks and the output nuclei of the basal ganglia; other regions implicated in gait and balance control, including the subthalamic nucleus, the external segment of the globus pallidus and the cerebellum, were therefore not examined and warrant investigation in future work.

### 4.3 Conclusion

In conclusion, rs‐FC between the left DLPFC and the hypothalamus and between the S1 and the GPi discriminate fallers from nonfallers in PD. These findings highlight potential targets for interventions aimed at reducing fall risk. Future work should focus on validating current findings in larger cohorts and exploring their utility as biomarkers for fall risk assessment and fall prevention strategies.

## Author Contributions

**Rodrigo Vitorio:** writing – review and editing, visualization, formal analysis, writing – original draft. **Korey P. Wylie:** investigation, writing – review and editing, conceptualization, data curation, methodology. **Justin W. Andrushko:** formal analysis, visualization, writing – review and editing, writing – original draft.

## Funding

The authors have nothing to report.

## Ethics Statement

The study procedures were conducted in accordance with the Declaration of Helsinki, and the study protocol was approved by the Colorado Multiple Institution Review Board.

## Consent

All participants gave their written consent for publication. All participants gave their written informed consent before they participated in the study.

## Conflicts of Interest

The authors declare no conflicts of interest.

## Data Availability

The structural and resting‐state functional MRI data analysed in this study are publicly available on OpenNeuro (https://openneuro.org/datasets/ds004392/versions/1.0.0). The clinical variables reported here (UPDRS‐III, Hoehn & Yahr stage, MoCA, LEDD and history of falls) are not included in the public release and were provided by K.P.W., an author of the original dataset. Derived connectivity measures and analysis code are available from the corresponding author on reasonable request.

## References

[ejn70666-bib-0001] Allen, N. E. , A. K. Schwarzel , and C. G. Canning . 2013. “Recurrent Falls in Parkinson's Disease: A Systematic Review.” Parkinson's Disease 2013: 906274. 10.1155/2013/906274 PMC3606768 23533953

[ejn70666-bib-0002] Araújo, H. , S. M. Smaili , R. Morris , et al. 2023. “Combination of Clinical and Gait Measures to Classify Fallers and Non‐Fallers in Parkinson's Disease.” Sensors (Basel) 23, no. 10: 4651. 37430565 10.3390/s23104651 PMC10221461

[ejn70666-bib-0003] Bianciardi, M. , C. Strong , N. Toschi , et al. 2018. “A Probabilistic Template of Human Mesopontine Tegmental Nuclei From In Vivo 7T MRI.” NeuroImage 170: 222–230. 28476663 10.1016/j.neuroimage.2017.04.070 PMC5670016

[ejn70666-bib-0004] Bianciardi, M. , N. Toschi , B. L. Edlow , et al. 2015. “Toward an In Vivo Neuroimaging Template of Human Brainstem Nuclei of the Ascending Arousal, Autonomic, and Motor Systems.” Brain Connectivity 5, no. 10: 597–607. 26066023 10.1089/brain.2015.0347 PMC4684653

[ejn70666-bib-0005] Cabrera‐Álvarez, J. , N. Doorn , F. Maestú , and G. Susi . 2023. “Modeling the Role of the Thalamus in Resting‐State Functional Connectivity: Nature or Structure.” PLoS Computational Biology 19, no. 8: e1011007. 37535694 10.1371/journal.pcbi.1011007 PMC10426958

[ejn70666-bib-0006] Desikan, R. S. , F. Ségonne , B. Fischl , et al. 2006. “An Automated Labeling System for Subdividing the Human Cerebral Cortex on MRI Scans Into Gyral Based Regions of Interest.” NeuroImage 31, no. 3: 968–980. 16530430 10.1016/j.neuroimage.2006.01.021

[ejn70666-bib-0007] Fan, L. , H. Li , J. Zhuo , et al. 2016. “The Human Brainnetome Atlas: A New Brain Atlas Based on Connectional Architecture.” Cerebral Cortex 26, no. 8: 3508–3526. 27230218 10.1093/cercor/bhw157 PMC4961028

[ejn70666-bib-0008] Fasano, A. , C. G. Canning , J. M. Hausdorff , S. Lord , and L. Rochester . 2017. “Falls in Parkinson's Disease: A Complex and Evolving Picture.” Movement Disorders: Official Journal of the Movement Disorder Society 32, no. 11: 1524–1536. 29067726 10.1002/mds.27195

[ejn70666-bib-0009] Florence, C. S. , G. Bergen , A. Atherly , E. Burns , J. Stevens , and C. Drake . 2018. “Medical Costs of Fatal and Nonfatal Falls in Older Adults.” Journal of the American Geriatrics Society 66, no. 4: 693–698. 29512120 10.1111/jgs.15304 PMC6089380

[ejn70666-bib-0010] Frazier, J. A. , S. Chiu , J. L. Breeze , et al. 2005. “Structural Brain Magnetic Resonance Imaging of Limbic and Thalamic Volumes in Pediatric Bipolar Disorder.” American Journal of Psychiatry 162, no. 7: 1256–1265. 15994707 10.1176/appi.ajp.162.7.1256

[ejn70666-bib-0011] García‐Gomar, M. G. , C. Strong , N. Toschi , et al. 2019. “In Vivo Probabilistic Structural Atlas of the Inferior and Superior Colliculi, Medial and Lateral Geniculate Nuclei and Superior Olivary Complex in Humans Based on 7 Tesla MRI.” Frontiers in Neuroscience 13: 764. 31440122 10.3389/fnins.2019.00764 PMC6694208

[ejn70666-bib-0012] García‐Gomar, M. G. , A. Videnovic , K. Singh , et al. 2022. “Disruption of Brainstem Structural Connectivity in REM Sleep Behavior Disorder Using 7 Tesla Magnetic Resonance Imaging.” Movement Disorders: Official Journal of the Movement Disorder Society 37, no. 4: 847–853. 34964520 10.1002/mds.28895 PMC9018552

[ejn70666-bib-0013] Gilat, M. , P. T. Bell , K. A. Ehgoetz Martens , et al. 2017. “Dopamine Depletion Impairs Gait Automaticity by Altering Cortico‐Striatal and Cerebellar Processing in Parkinson's Disease.” NeuroImage 152: 207–220. 28263926 10.1016/j.neuroimage.2017.02.073

[ejn70666-bib-0014] Goldstein, J. M. , L. J. Seidman , N. Makris , et al. 2007. “Hypothalamic Abnormalities in Schizophrenia: Sex Effects and Genetic Vulnerability.” Biological Psychiatry 61, no. 8: 935–945. 17046727 10.1016/j.biopsych.2006.06.027

[ejn70666-bib-0015] Gorst, T. , J. Marsden , and J. Freeman . 2019. “Lower Limb Somatosensory Discrimination Is Impaired in People With Parkinson's Disease: Novel Assessment and Associations With Balance, Gait, and Falls.” Movement Disorders Clinical Practice 6, no. 7: 593–600. 31538094 10.1002/mdc3.12831 PMC6749802

[ejn70666-bib-0016] Griffanti, L. , G. Salimi‐Khorshidi , C. F. Beckmann , et al. 2014. “ICA‐Based Artefact Removal and Accelerated fMRI Acquisition for Improved Resting State Network Imaging.” NeuroImage 95: 232–247. 24657355 10.1016/j.neuroimage.2014.03.034 PMC4154346

[ejn70666-bib-0017] Hamacher, D. , F. Herold , P. Wiegel , D. Hamacher , and L. Schega . 2015. “Brain Activity During Walking: A Systematic Review.” Neuroscience and Biobehavioral Reviews 57: 310–327. 26306029 10.1016/j.neubiorev.2015.08.002

[ejn70666-bib-0018] Herold, F. , P. Wiegel , F. Scholkmann , A. Thiers , D. Hamacher , and L. Schega . 2017. “Functional Near‐Infrared Spectroscopy in Movement Science: A Systematic Review on Cortical Activity in Postural and Walking Tasks.” Neurophotonics 4, no. 4: 041403. 28924563 10.1117/1.NPh.4.4.041403 PMC5538329

[ejn70666-bib-0019] Hosmer, D. W. , S. Lemeshow , and R. X. Sturdivant . 2013. Applied Logistic Regression. 3rd ed. John Wiley & Sons.

[ejn70666-bib-0020] Hughes, A. J. , S. E. Daniel , L. Kilford , and A. J. Lees . 1992. “Accuracy of Clinical Diagnosis of Idiopathic Parkinson's Disease: A Clinico‐Pathological Study of 100 Cases.” Journal of Neurology, Neurosurgery, and Psychiatry 55, no. 3: 181–184. 1564476 10.1136/jnnp.55.3.181 PMC1014720

[ejn70666-bib-0021] Jenkinson, M. , P. Bannister , M. Brady , and S. Smith . 2002. “Improved Optimization for the Robust and Accurate Linear Registration and Motion Correction of Brain Images.” NeuroImage 17, no. 2: 825–841. 12377157 10.1016/s1053-8119(02)91132-8

[ejn70666-bib-0022] Jenkinson, M. , C. F. Beckmann , T. E. Behrens , M. W. Woolrich , and S. M. Smith . 2012. “FSL.” NeuroImage 62, no. 2: 782–790. 21979382 10.1016/j.neuroimage.2011.09.015

[ejn70666-bib-0023] Jenkinson, M. , and S. Smith . 2001. “A Global Optimisation Method for Robust Affine Registration of Brain Images.” Medical Image Analysis 5, no. 2: 143–156. 11516708 10.1016/s1361-8415(01)00036-6

[ejn70666-bib-0024] Kaller, C. P. , B. Rahm , J. Spreer , C. Weiller , and J. M. Unterrainer . 2011. “Dissociable Contributions of Left and Right Dorsolateral Prefrontal Cortex in Planning.” Cerebral Cortex 21, no. 2: 307–317. 20522540 10.1093/cercor/bhq096

[ejn70666-bib-0025] la Fougere, C. , A. Zwergal , A. Rominger , et al. 2010. “Real Versus Imagined Locomotion: A [^18^F]‐FDG PET‐fMRI Comparison.” NeuroImage 50, no. 4: 1589–1598. 20034578 10.1016/j.neuroimage.2009.12.060

[ejn70666-bib-0026] Lanciego, J. L. , N. Luquin , and J. A. Obeso . 2012. “Functional Neuroanatomy of the Basal Ganglia.” Cold Spring Harbor Perspectives in Medicine 2, no. 12: a009621. 23071379 10.1101/cshperspect.a009621 PMC3543080

[ejn70666-bib-0027] Love, J. , R. Selker , M. Marsman , et al. 2019. “JASP: Graphical Statistical Software for Common Statistical Designs.” Journal of Statistical Software 88, no. 2: 1–17.

[ejn70666-bib-0028] Maidan, I. , H. Bernad‐Elazari , E. Gazit , N. Giladi , J. M. Hausdorff , and A. Mirelman . 2015. “Changes in Oxygenated Hemoglobin Link Freezing of Gait to Frontal Activation in Patients With Parkinson Disease: An fNIRS Study of Transient Motor‐Cognitive Failures.” Journal of Neurology 262, no. 4: 899–908. 25636682 10.1007/s00415-015-7650-6

[ejn70666-bib-0029] Makris, N. , J. M. Goldstein , D. Kennedy , et al. 2006. “Decreased Volume of Left and Total Anterior Insular Lobule in Schizophrenia.” Schizophrenia Research 83, no. 2–3: 155–171. 16448806 10.1016/j.schres.2005.11.020

[ejn70666-bib-0030] Orcioli‐Silva, D. , R. Vitório , V. S. Beretta , et al. 2021. “Is Cortical Activation During Walking Different Between Parkinson's Disease Motor Subtypes?” Journals of Gerontology Series A: Biological Sciences and Medical Sciences 76, no. 4: 561–567. 32674140 10.1093/gerona/glaa174

[ejn70666-bib-0031] Orcioli‐Silva, D. , R. Vitório , P. Nóbrega‐Sousa , et al. 2020. “Levodopa Facilitates Prefrontal Cortex Activation During Dual Task Walking in Parkinson Disease.” Neurorehabilitation and Neural Repair 34, no. 7: 589–599. 32449460 10.1177/1545968320924430

[ejn70666-bib-0032] Otomune, H. , M. Mihara , N. Hattori , et al. 2019. “Involvement of Cortical Dysfunction in Frequent Falls in Patients With Parkinson's Disease.” Parkinsonism & Related Disorders 64: 169–174. 30992233 10.1016/j.parkreldis.2019.04.007

[ejn70666-bib-0033] Patenaude, B. , S. M. Smith , D. N. Kennedy , and M. Jenkinson . 2011. “A Bayesian Model of Shape and Appearance for Subcortical Brain Segmentation.” NeuroImage 56, no. 3: 907–922. 21352927 10.1016/j.neuroimage.2011.02.046 PMC3417233

[ejn70666-bib-0034] Paul, S. S. , L. Harvey , C. G. Canning , et al. 2017. “Fall‐Related Hospitalization in People With Parkinson's Disease.” European Journal of Neurology 24, no. 3: 523–529. 28117538 10.1111/ene.13238

[ejn70666-bib-0035] Permezel, F. , J. Alty , I. H. Harding , and D. Thyagarajan . 2023. “Brain Networks Involved in Sensory Perception in Parkinson's Disease: A Scoping Review.” Brain Sciences 13, no. 11: 1552. 38002513 10.3390/brainsci13111552 PMC10669548

[ejn70666-bib-0036] Prodoehl, J. , H. Yu , D. M. Little , I. Abraham , and D. E. Vaillancourt . 2008. “Region of Interest Template for the Human Basal Ganglia: Comparing EPI and Standardized Space Approaches.” NeuroImage 39, no. 3: 956–965. 17988895 10.1016/j.neuroimage.2007.09.027 PMC2253186

[ejn70666-bib-0037] Ragothaman, A. , M. Mancini , J. G. Nutt , D. A. Fair , O. Miranda‐Dominguez , and F. B. Horak . 2022. “Resting State Functional Networks Predict Different Aspects of Postural Control in Parkinson's Disease.” Gait & Posture 97: 122–129. 35931013 10.1016/j.gaitpost.2022.07.003

[ejn70666-bib-0038] Rosenberg‐Katz, K. , T. Herman , Y. Jacob , et al. 2015. “Fall Risk Is Associated With Amplified Functional Connectivity of the Central Executive Network in Patients With Parkinson's Disease.” Journal of Neurology 262, no. 11: 2448–2456. 26233691 10.1007/s00415-015-7865-6

[ejn70666-bib-0039] Rudzińska, M. , S. Bukowczan , J. Stożek , et al. 2013. “Causes and Consequences of Falls in Parkinson Disease Patients in a Prospective Study.” Neurologia i Neurochirurgia Polska 47, no. 5: 423–430. 24166563 10.5114/ninp.2013.38222

[ejn70666-bib-0040] Salimi‐Khorshidi, G. , G. Douaud , C. F. Beckmann , M. F. Glasser , L. Griffanti , and S. M. Smith . 2014. “Automatic Denoising of Functional MRI Data: Combining Independent Component Analysis and Hierarchical Fusion of Classifiers.” NeuroImage 90: 449–468. 24389422 10.1016/j.neuroimage.2013.11.046 PMC4019210

[ejn70666-bib-0041] Sherman, S. M. , and R. W. Guillery . 2002. “The Role of the Thalamus in the Flow of Information to the Cortex.” Philosophical Transactions of the Royal Society of London. Series B, Biological Sciences 357, no. 1428: 1695–1708. 12626004 10.1098/rstb.2002.1161 PMC1693087

[ejn70666-bib-0042] Singh, K. , M. G. García‐Gomar , and M. Bianciardi . 2021. “Probabilistic Atlas of the Mesencephalic Reticular Formation, Isthmic Reticular Formation, Microcellular Tegmental Nucleus, Ventral Tegmental Area Nucleus Complex, and Caudal‐Rostral Linear Raphe Nucleus Complex in Living Humans From 7 Tesla Magnetic Resonance Imaging.” Brain Connectivity 11, no. 8: 613–623. 33926237 10.1089/brain.2020.0975 PMC8817713

[ejn70666-bib-0043] Singh, K. , I. Indovina , J. C. Augustinack , et al. 2019. “Probabilistic Template of the Lateral Parabrachial Nucleus, Medial Parabrachial Nucleus, Vestibular Nuclei Complex, and Medullary Viscero‐Sensory‐Motor Nuclei Complex in Living Humans From 7 Tesla MRI.” Frontiers in Neuroscience 13: 1425. 32038134 10.3389/fnins.2019.01425 PMC6989551

[ejn70666-bib-0044] Smith, S. M. 2002. “Fast Robust Automated Brain Extraction.” Human Brain Mapping 17, no. 3: 143–155. 12391568 10.1002/hbm.10062 PMC6871816

[ejn70666-bib-0045] Smith, S. M. , M. Jenkinson , M. W. Woolrich , et al. 2004. “Advances in Functional and Structural MR Image Analysis and Implementation as FSL.” NeuroImage 23, no. Suppl 1: S208–S219. 15501092 10.1016/j.neuroimage.2004.07.051

[ejn70666-bib-0046] Takakusaki, K. 2017. “Functional Neuroanatomy for Posture and Gait Control.” Journal of Movement Disorders 10, no. 1: 1–17. 28122432 10.14802/jmd.16062 PMC5288669

[ejn70666-bib-0047] Vitório, R. , E. Lirani‐Silva , D. Orcioli‐Silva , V. S. Beretta , A. S. Oliveira , and L. T. B. Gobbi . 2023. “Electrocortical Dynamics of Usual Walking and the Planning to Step Over Obstacles in Parkinson's Disease.” Sensors 23, no. 10: 4866. 37430780 10.3390/s23104866 PMC10224292

[ejn70666-bib-0048] Vitorio, R. , M. Mancini , P. Carlson‐Kuhta , F. B. Horak , and V. V. Shah . 2023. “Should We Use Both Clinical and Mobility Measures to Identify Fallers in Parkinson's Disease?” Parkinsonism & Related Disorders 106: 105235. 36512851 10.1016/j.parkreldis.2022.105235 PMC10756255

[ejn70666-bib-0049] Wang, Y. , T. Gong , N. Tao , et al. 2024. “Hypo‐Connectivity of the Primary Somatosensory Cortex in Parkinson's Disease: A Resting‐State Functional MRI Study.” Frontiers in Neurology 15: 1361063. 38746656 10.3389/fneur.2024.1361063 PMC11091379

[ejn70666-bib-0050] Woolrich, M. W. , S. Jbabdi , B. Patenaude , et al. 2009. “Bayesian Analysis of Neuroimaging Data in FSL.” NeuroImage 45: S173–S186. 19059349 10.1016/j.neuroimage.2008.10.055

[ejn70666-bib-0051] Worden, R. , M. S. Bennett , and V. Neacsu . 2021. “The Thalamus as a Blackboard for Perception and Planning.” Frontiers in Behavioral Neuroscience 15: 633872. 33732119 10.3389/fnbeh.2021.633872 PMC7956969

[ejn70666-bib-0052] Wylie, K. P. , B. M. Kluger , L. D. Medina , et al. 2023. “Hippocampal, Basal Ganglia and Olfactory Connectivity Contribute to Cognitive Impairments in Parkinson's Disease.” European Journal of Neuroscience 57, no. 3: 511–526. 36516060 10.1111/ejn.15899 PMC9970048

[ejn70666-bib-0053] Wylie, K. P. , B. M. Kluger , L. D. Medina , et al. 2026. “Parkinson's Disease, Functional Connectivity, and Cognition.” OpenNeuro. 10.18112/openneuro.ds004392.v1.0.2.

[ejn70666-bib-0054] Zhang, Y. , M. Brady , and S. Smith . 2001. “Segmentation of Brain MR Images Through a Hidden Markov Random Field Model and the Expectation‐Maximization Algorithm.” IEEE Transactions on Medical Imaging 20, no. 1: 45–57. 11293691 10.1109/42.906424

[ejn70666-bib-0055] Zigmond, A. S. , and R. P. Snaith . 1983. “The Hospital Anxiety and Depression Scale.” Acta Psychiatrica Scandinavica 67, no. 6: 361–370. 6880820 10.1111/j.1600-0447.1983.tb09716.x

